# Advance directives, proxy opinions, and treatment restrictions in patients with severe stroke

**DOI:** 10.1186/s12904-017-0234-8

**Published:** 2017-11-14

**Authors:** Floor A. S. de Kort, Marjolein Geurts, Paul L. M. de Kort, Julia H. van Tuijl, Ghislaine J. M. W. van Thiel, L. Jaap Kappelle, H. Bart van der Worp

**Affiliations:** 1 Department of Neurology and Neurosurgery, Brain Center Rudolf Magnus, University Medical Center Utrecht, Utrecht, the Netherlands; 2 Department of Neurology, Elisabeth-Twee Steden ziekenhuis, Tilburg, the Netherlands; 3 Julius Center for Health Sciences and Primary Care, University Medical Center Utrecht, Utrecht, the Netherlands

**Keywords:** Stroke, Ethics, End-of-life decisions, Advance care planning, Advance directives, Proxy opinions, Surrogate decision making

## Abstract

**Background:**

Patients with severe stroke often do not have the capacity to participate in discussions on treatment restrictions because of a reduced level of consciousness, aphasia, or another cognitive disorder. We assessed the role of advance directives and proxy opinions in the decision-making process of incapacitated patients.

**Methods:**

Sixty patients with severe functional dependence (Barthel Index ≤6) at day four after ischemic stroke or intracerebral hemorrhage were included in a prospective two-center cohort study. The decision-making process with respect to treatment restrictions was assessed by means of a semi-structured questionnaire administered to the treating physician at the day of inclusion.

**Results:**

Forty-nine patients (82%) did not have the capacity to participate in the decision-making process. In eight patients, there was no discussion on treatment restrictions and full care was installed. In 41 patients, the decision whether to install treatment restrictions was discussed with proxies. One patient had a written advance directive. In the remaining 40 patients, proxies based their opinion on previously expressed wishes of the patient (18 patients) or advised in the best interest of the patient (22 patients). In 36 of 41 patients, treatment restrictions were installed after agreement between physician and proxy. At six months, 23 of 49 patients had survived. In only three of them the decision on treatment restrictions was based on previously expressed wishes. Remarkably, two of these survivors could not recall any of their alleged previously expressed wishes.

**Conclusions:**

Treatment restrictions were installed in the majority of incapacitated patients after stroke. Proxy opinions frequently served as the best way to respect the patients’ autonomy, but their accuracy remains unclear.

**Electronic supplementary material:**

The online version of this article (10.1186/s12904-017-0234-8) contains supplementary material, which is available to authorized users.

## Background

Patients with severe stroke have a high risk of long-term disability or death. A substantial proportion of in-hospital deaths after severe stroke occur in the context of withholding or withdrawal of life prolonging treatments [1, 2]. The decision to forgo life prolonging treatment usually evolves from discussions that are complicated by several factors. First, in contrast to more chronic diseases, stroke occurs almost always unexpectedly. Secondly, prognosis is often uncertain in the early stage. Thirdly, continuation of treatment may allow patients to survive for months or years, at the cost of being left in a state of disability that might be against their wishes [3]. Fourthly, patients with severe stroke often do not have the capacity to participate in the decision-making process on treatment restrictions themselves because of a reduced level of consciousness, aphasia, or another cognitive disorder [3]. In these cases, proxy opinions and advance directives are used to warrant patients’ autonomy [4].

The principle of autonomy is considered one of the fundamental principles of bioethics in Western societies and it plays a major role in modern health care systems [5]. Respect for the autonomy of the patient implies that the patient has the capacity to decide intentionally, with understanding, and without controlling influences that would mitigate against a free and voluntary decision [5]. A direct translation of respect for patients’ autonomy is the doctrine of informed consent. For most treatments, explicit informed consent is required and a capacitated patient has the right to refuse treatment. In The Netherlands, among other countries such as Belgium, Denmark and Canada, the requirement of informed consent is embedded in the law [6]. In incapacitated patients, a proxy decision maker has to be identified. This legal representative of the patient has to consent to treatment on his behalf. Dutch law states that the patient can be represented by several proxies in descending order: an appointed guardian; an individual to whom the patient has given a durable power of attorney that includes the authority to make health care decisions; the patient’s spouse or registered domestic partner; children of the patient who are at least eighteen years of age, parents of the patient or adult brothers and sisters of the patient [6]. Advance directives can help to respect the patient’s (former) autonomy. A written non treatment directive has a strong legal status in the Netherlands: in principle, it equals the current refusal of a capacitated patient [6]. The law does not distinguish between self-created advance directives or specific documents for healthcare decision-making. Advance directives are not nationally registered in the Netherlands and are usually kept by the patient and/or by his general practitioner.

Although discussions about treatment restrictions are routine in the care for patients with severe stroke in many countries, it is unclear how physicians implement advance directives and proxy opinions in these discussions. In this study, we assessed current practices in the decision whether or not to install treatment restrictions in incapacitated patients with severe stroke.

## Methods

We selected patients from the ‘Advance Directives And Proxy opinions in acute sTroke’ (ADAPT) cohort [7], a prospective two-center cohort study which included consecutive patients admitted at the stroke unit with acute severe ischemic stroke or intracerebral hemorrhage and a very small chance of functional independency after 6 months, defined as Barthel Index (BI) ≤6 out of 20 at day 4 [8]. Patients with subarachnoid hemorrhage and patients without an available legal representative were excluded from the study. Patients were included between September 2012 and December 2013 in the University Medical Center Utrecht, and between January and December 2013 in the St. Elisabeth hospital in Tilburg, a large regional teaching hospital in The Netherlands. The main aim of the ADAPT study was to assess the association between the placement of treatment restrictions and mortality in patients who had survived the first four days after severe ischemic stroke or intracerebral hemorrhage. The original cohort study included 60 patients [7].

For this secondary study, all patients whom their treating physicians considered incapacitated to participate in discussions on treatment restrictions were selected (49 patients). The judgment of the patient’s decision-making capacity was based on a clinical assessment using the internationally accepted definition of capacity: understanding, expressing a choice, appreciation, and reasoning [9].

The legal representative appointed conformed with Dutch law as described in the Introduction. The study was approved by the institutional review board of each center and written informed consent was obtained from each patient or a legal representative.

### Data collection

Demographic and stroke characteristics were collected from the patients’ charts. The decision-making process concerning the instalment of treatment restrictions was assessed by a semi-structured questionnaire administered to the treating physician at the day of the patient’s inclusion. The questionnaire included both open-ended and closed-ended questions on the following items:

1. Physician’s judgment of the decision-making capacity of the patient whether or not to install treatment restrictions.
2. The presence and content of advance directives. Identification of advance directives was left to the responsibility of the treating physician.
3. The role of proxies in the decision-making process, as perceived by the treating physician.
4. Factors that contributed to the instalment of treatment restrictions.

### Follow-up

One investigator (FASdK) visited each patient who had survived and their caregiver at six months (+/− six weeks) after stroke. Their reflection on the decision-making process, including the presence of advance directives, was assessed by a semi-structured questionnaire (Additional file 1). All questionnaires were analyzed by hand; coding verbatim was not used.

## Results

Of 60 patients included in ADAPT [7], 49 (82%) patients were, according to their physician, incapacitated to decide whether or not to install treatment restrictions. The reasons for incapacity were a reduced level of consciousness in 14 (29%), aphasia in 10 (20%), cognitive impairment in 6 (12%), or a combination of two or more of these conditions in 19 (39%) cases. Twenty-one treating physicians filled out the questionnaires.

The median time between stroke onset and inclusion was 6 days (range, 4-10). The mean age of the patients was 72 years (SD 15); 26 (53%) were male; 27 (55%) had an ischemic stroke; the median National Institutes of Health Stroke Scale (NIHSS) score on admission was 18 (range, 12-21), and the median BI at day 4 was 0 (range, 0-2).

### Treatment restrictions

**Fig. 1:**
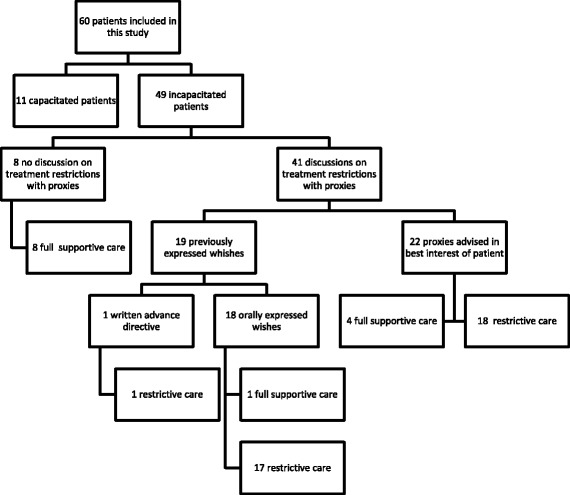
Flow of patients through this study

In 36 of 49 incapacitated patients (74%), treatment restrictions had been installed at the time of study inclusion (Fig. 1). The remaining 13 patients received full care. Reasons for the decision whether or not to install treatment restrictions are summarized in Table 1. Table 2 shows the type of treatment restrictions installed.

**Table 1:** Physicians’ reasons for restrictive or full care (more than one option possible)

	Incapacitated patients with restrictive care (*n* = 36)	Incapacitated patients with full care (*n* = 13)	
		Patients without discussions on treatment restrictions (*n* = 8)	Patients with discussions on treatment restrictions (*n* = 5)
Proxy and/or patient preferences, n(%)	14 (39)	NA	3 (60)
Physicians’ estimate of functional recovery, n(%)	32 (89)	7 (88)	3 (60)
Age, n(%)	24 (67)	7 (88)	1 (20)
Comorbidity, n(%)	18 (50)	0 (0)	2 (40)
Discomfort, n(%)	5 (14)	0 (0)	0 (0)
Religion, n(%)	0 (0)	0 (0)	1 (20)

**Table 2:** Decisions on treatment restrictions in incapacitated patients

	Patients without discussions on treatment restrictions (*n* = 8)	Patients with discussions on treatment restrictions (*n* = 41)	
		Patients who previously expressed their wishes (written/oral) (*n* = 19)	Patients in whom was advised in their best interest (*n* = 22)
Full supportive care	8	1	4
DNR	0	4	6
Withhold admission at ICU	0	6	9
No curative treatment of complications	0	1	0
Withhold artificial nutrition and hydration	0	7	3

*DNR* Do not resuscitate, *ICU* Intensive Care Unit

### Discussions on treatment restrictions

In 8 of 49 (16%) incapacitated patients, a discussion on treatment restrictions had not taken place, the treating physician had decided on full care in these cases (Fig. 1). Reasons not to discuss treatment restrictions were a young age (88%) and the physician’s expectation of a good functional recovery (88%) (Table 1). In the remaining 41 patients, treatment restrictions were discussed. Nineteen of 41 patients had previously expressed their wishes (one patient had a written advance directive and 18 of them had orally expressed their wishes) (Fig. 1, Table 2).

### Advance directives and proxy opinions

In 41 of 49 incapacitated patients (82%), the decision on treatment restrictions was discussed with the patients’ proxies. One patient had a written advance directive requesting restrictive care in case of dependency, a “do not resuscitate-order” was installed. In the remaining 40 patients, the decision whether or not to install treatment restrictions was informed by proxy opinions. Proxy opinions were either based on previously expressed wishes of the patient (18 patients, resulting in restrictive care in 17 (94%) cases), or based on the perceived best interest of the patient in the absence of such previous expressions (22 patients, resulting in restrictive care in 18 patients (82%) and full care in four (18%) (Fig. 1).

### Follow up

At six months, 23 (47%) patients who were incapacitated at the time of the discussion on treatment restrictions had survived. Fifteen of them (65%) had a poor functional outcome.

Six of eight patients in whom no discussion on treatment restrictions had taken place survived. All six patients had received full care after stroke. At six months, five of them retrospectively agreed with this decision.

The single patient with a written advance directive was one of the survivors at six months. This patient still agreed on the content of his advance directive (restrictive care in case of dependency). At follow-up, none of the patients stated they had a written advance directive that was missed in the acute stage.

Only three of 18 patients for whom treatment decisions were discussed with proxies and were based on previously expressed wishes, survived up to six months. Remarkably, two of these survivors could not recall any of their alleged previously expressed wishes.

Of the 22 patients for whom proxies had advised in their best interest without known previously expressed wishes, 13 (59%) survived up to six months. Four of them could not complete the interview at six months, three because of aphasia and one patient was moribund at the time of follow-up. The remaining nine survivors retrospectively agreed with the decisions on treatment restrictions made in the early phase of their stroke. One of nine patients stated he orally expressed wishes about treatment to proxies before the stroke; this expression was not reported by the treating physician.

## Discussion

This study shows that in incapacitated patients with a very recent stroke, discussions on treatment restrictions are complex. Advance directives are scarce. Patients’ autonomy is mostly respected via proxies, who base their opinion on previously expressed wishes of the patient or advised in the best interest of the patient. This mostly resulted in restrictive care. It remained unclear whether proxies adequately reflected the patients’ preferences. In a substantial proportion (16%) of incapacitated patients, no discussion on treatment restrictions between treating physicians and patients or their proxies had taken place.

In our study, an advance directive was available in only one out of 49 patients. This is most likely the consequence of the acute course of the disease, in combination with a low prevalence of advance directives in the general population [10]. Population studies in The Netherlands show that only 7% of the general population has completed an advance directive [11]. The prevalence of advance directives in advanced stages of cancer has been estimated about 55% [12, 13]. Most advance directives are written in the last days of life, which suggests that disease itself is an important reason to write an advance directive [14]. The value of advance directives can be limited [3] as they often relate to very specific situations such as coma, and applying these wishes to a situation in which the patient has a focal deficit caused by stroke might not be appropriate [15, 16]. In a German observational cohort study, less than half of the available advance directives were considered applicable in patients suffering severe acute stroke [17].

In case of incapacity of the patient to participate in the decision-making process, the treating physician should discuss the condition of the patient with a legal representative, usually a family member. In many countries, legal representatives have a strong legal status [18]. In clinical practice, advance directives and proxy opinions are equally effective in influencing doctors’ decisions [19]. Vignette studies show that physicians have a mild preference to forgo life sustaining treatment in case of contradictions between written advance directives and proxy opinion [20]. In our study, two of the three patients in whom a proxy opinion was based on alleged previously expressed wishes, and who could be interviewed at six months, could not recall this expression. Although the number is very small, it raises questions about the accuracy of proxy opinions. These findings are in line with the accuracy of surrogate decisions observed in hypothetical scenario studies [21]. Legal representatives may be affected by their own stress and by distraction from familial or social factors and recall bias [3].

Two factors further complicate the use of advance directives and proxy opinions in acute stroke patients. First, the ‘disability paradox’: the fact that patients often report greater happiness and quality of life than healthy people predict they would under the same circumstances [22]. Second, the ‘response shift’: a change of internal standards, values and the conceptualization of quality of life as a result of changes in health status [23]. A considerable proportion of patients after severe stroke recaptures a good quality of life despite severe disability [24]. It is hard to identify patients in the acute stage after stroke who might adapt well to their new situation [22, 25, 26].

In current practice, implementing patient centeredness of care in acute severe stroke is challenging. Efforts should be made to improve individualized end-of-life decision-making. The prevalence of advance directives in the general population can be increased by advance care planning campaigns on the community level, such as the ‘Speak Up’-campaign in Canada [27] and the ‘Advance Care Planning Australia’-initiative [28]. In these campaigns, special attention should be drawn to stroke scenarios to improve the applicability of advance directives in severe stroke. However, in the light of the phenomena of response shift and the disability paradox, the predictive value of treatment directives in stroke in general should not be overestimated. A joint assessment of the physician and the proxy with regard to the patient’s best interest at the moment of actual decision-making on life sustaining treatments should always be put into the equation.

As treatment restrictions are independently associated with mortality [7], decisions on withholding or withdrawal of life-sustaining treatments should be taken with great caution. The treating physician carries the final responsibility for medical treatment decisions in incapacitated patients. It can be an enormous emotional burden for legal representatives to feel the sole responsibility for treatment restrictions and it is therefore essential to avoid giving families the impression they are being asked to make these major decisions on their own [29–31].

To implement patient preferences in the decision-making process, we previously introduced a 5-step approach [3]. The first step is collection of evidence, in which the treating physician defines clinical problems and outweighs the risks and benefits of withdrawal or continuation of specific medical treatments. Second, the physician shares information with legal representatives, in which he/she explains the clinical problems and expected prognosis, and sketches scenarios in which specific medical treatment is withdrawn or given. A crucial part of this step is that legal representatives share patient preferences and values with the physician. Third, the physician critically appraises the collected information and addresses biases of both prognostication and patient preferences that could influence outcome. Fourth, the physician makes a recommendation and promotes shared-decision making. Finally, the physician provides adequate follow-up.

### Limitations

This study has limitations. The sample size prohibits strong generalization of our findings. We included patients who were severely dependent but still alive at day four, because treatment restrictions are most often considered in these patients [15]. Therefore, our results cannot be extrapolated to situations at different points in time after stroke or in patients who are less severely disabled at day four, and we may have missed discussions and decisions on treatment restrictions at later stages. The data are relatively old (inclusion started in September 2012), but there is no reason to assume that this affects our findings. Furthermore, information on the decision-making process was obtained from the treating physician and therefore reflects the physician’s vision on patient preferences. However, this appears appropriate because it is the physician who finally makes the decision to withhold or continue specific treatments. At six-months follow-up, recall bias might have played a role, which may have led to more positive reflection on the process, and patients might have given desirable answers on questions about treatment limitations during home-visits. Finally, we did not contact proxies of deceased patients.

## Conclusions

Our study shows that advance directives are scarce in patients with a major disabling stroke who cannot participate in the discussion whether or not treatment restrictions should be installed. Proxy opinions are frequently used as a way to respect the patients’ autonomy, but the treating physician should be cautious not to overestimate the capability of these proxies to reflect the opinion of the patient as based on previously expressed wishes.

## Additional file

Additional file 1: Questionnaire patient 6 months after stroke. (PDF 407 kb)

## Abbreviations

ADAPT: Advance Directives And Proxy opinions in acute sTroke
BI: Barthel Index
NIHSS: National Institutes of Health Stroke Scale
