# Supplementary material for: Advance directives, proxy opinions, and treatment restrictions in patients with severe stroke
Source: BMC Palliat Care. 2017 Nov 14;16:52. doi: 10.1186/s12904-017-0234-8 (PMC5686946; doi:10.1186/s12904-017-0234-8)
Supplement: Additional file 1: — Questionnaire patient 6 months after stroke. (PDF 407 kb) [file 12904_2017_234_MOESM1_ESM.pdf]

QUESTIONNAIRE PATIENT 6 MONTHS AFTER STROKE

1. Current situation

a. How would you value your life in the last week?

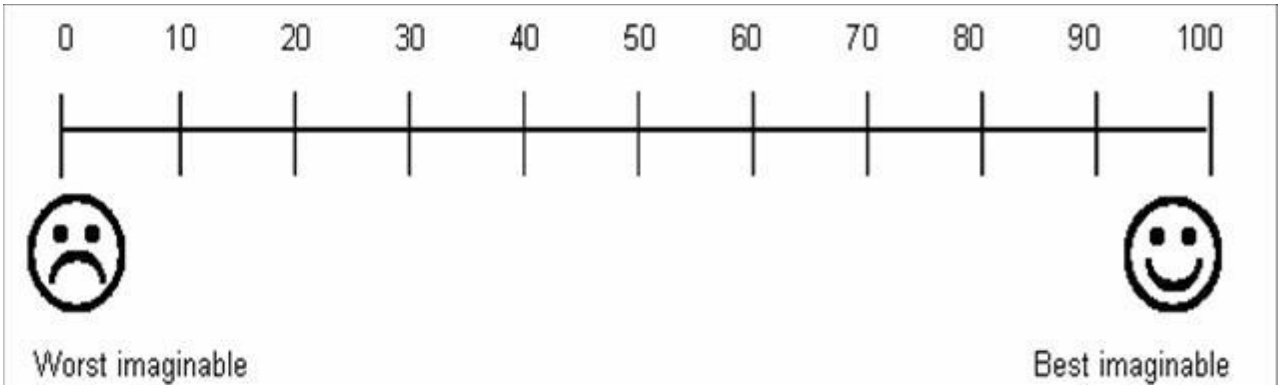

2. Situation prior to stroke

a. Please think back at your situation prior to stroke. You might have had an idea about how you would value life after stroke. Could you tell us how good or bad you thought life after stroke would be?

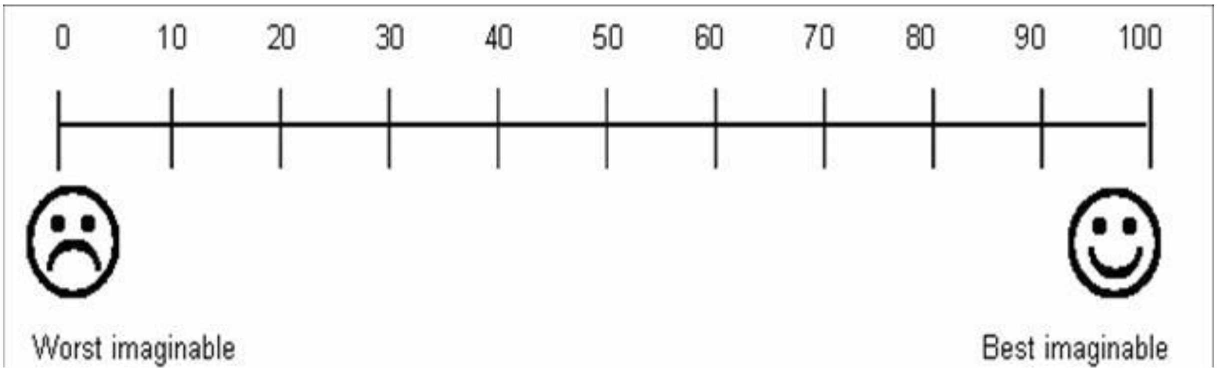

b. Did you have a written advance directive prior to stroke?

☐ Yes, namely:

☐ No

☐ I don't know

c. Did you, prior to stroke, ever had a conversation with your proxies about what your wishes would be after stroke?

☐ Yes, with:

☐ No

d. If yes, what where your wishes?

e. What where your thoughts about a stroke prior to your own stroke?

☐ I thought the consequences of stroke were less severe

☐ I thought the consequences of stroke were exactly as they are now

☐ I thought the consequences of stroke were more severe

☐ I don’t know

f. If you, prior to stroke, had known about your current situation, would your advance directives or the conversation you had with your proxies about your wishes have been different?

☐ Yes (go to question g)

☐ No (go to question 3)

☐ I don’t know

g. What would your conclusion, in retrospect, have been then?

☐ I would have worshipped life after stroke more and I would therefore have opted for full care

☐ I would have worshipped life after stroke less and I would therefore have opted for less aggressive care

☐ No conclusions

☐ I don’t know

☐ Other, namely:

3. Situation directly after stroke

a. Please think back at the first week after your stroke. How would you, in retrospect, rate your quality of life at that time?

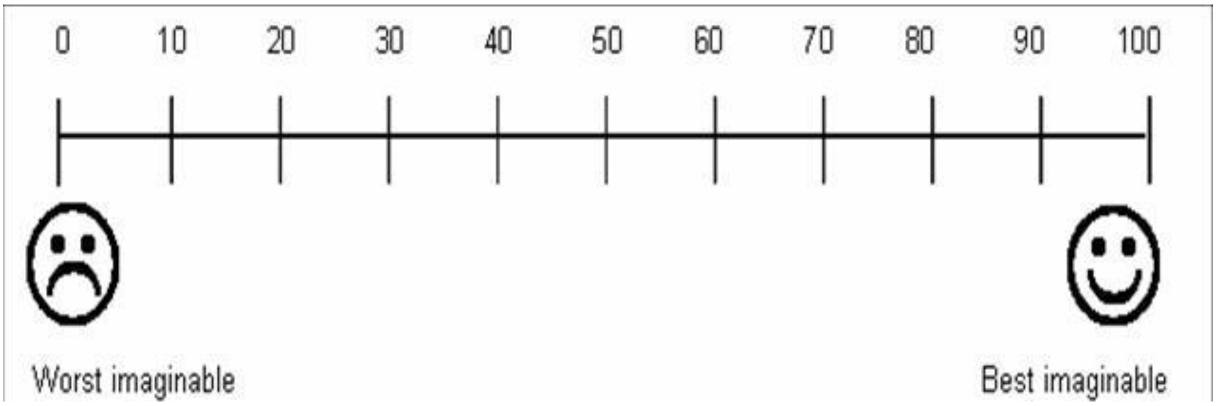

4. Decision-making process

How do you look back at the decision-making process regarding end-of-life decisions after your stroke?
